# Association of Gestational Weight Gain With Preterm Birth Among Women of Normal Pre-pregnancy Body Mass Index: A Matched Case-Controlled Study

**DOI:** 10.7759/cureus.104917

**Published:** 2026-03-09

**Authors:** Mounika Itha, Bhabani Pegu, Battini VijayaLakshmi, Nishaant Ramasamy, Anish Keepanasseril

**Affiliations:** 1 Obstetrics and Gynaecology, Jawaharlal Institute of Postgraduate Medical Education and Research, Puducherry, IND; 2 Community Medicine/Preventive Medicine, All India Institute of Medical Sciences, Nagpur, Nagpur, IND

**Keywords:** dietary habits, gestational weight gain, normal body mass index, pregnancy outcomes, preterm birth

## Abstract

Background: Preterm birth (PTB), i.e., delivery before 37 weeks, is a major cause of neonatal morbidity and mortality. Gestational weight gain (GWG) influences PTB risk, with both inadequate and excessive gain linked to adverse outcomes.

Objective: To assess the association between GWG and PTB among women with normal pre-pregnancy BMI.

Methods: A matched case-control study was conducted at the Women and Children’s Hospital, Jawaharlal Institute of Postgraduate Medical Education and Research, India. Women with spontaneous preterm delivery (28-36 weeks; n = 90) were matched in a ratio of 1:4 by age (±2 years) and parity with term controls (n = 360). GWG was calculated as the difference between pre-pregnancy and delivery weight and categorised per the Institute of Medicine (IOM) guidelines as inadequate, adequate, and excessive. Maternal sociodemographic, obstetric, and clinical data were collected from records and interviews. Conditional logistic regression evaluated associations between GWG and PTB, adjusting for confounders.

Results: Cases had a lower mean GWG than controls (6.77 ± 2.17 vs. 8.91 ± 1.86 kg, p < 0.001) but delivered six weeks earlier, limiting weight gain. After adjusting for gestational age, GWG was not independently associated with PTB. Higher maternal education was protective (OR: 0.41; 95% CI: 0.18-0.98), whereas prior caesarean increased risk (OR: 5.23; 95% CI: 1.87-14.67).

Conclusion: Extremes of GWG were not independently associated with PTB in women with normal BMI. Higher maternal education was protective, whereas prior caesarean increased risk. Monitoring GWG and promoting maternal health literacy may help prevent preterm deliveries.

## Introduction

Preterm birth (PTB), defined as delivery before 37 completed weeks of gestation, remains a major contributor to neonatal morbidity and mortality worldwide, affecting approximately 10% of all births [1,2]. Identifying modifiable maternal factors associated with PTB is therefore essential for improving pregnancy outcomes. Gestational weight gain (GWG) is one such factor that plays an important role in maternal health and fetal growth [3]. Current guidelines from the Institute of Medicine (IOM) recommend a GWG of 11.5-16 kg for women with a normal pre-pregnancy body mass index (BMI) [4]. However, adherence to these recommendations is often suboptimal, and both inadequate and excessive GWG have been associated with adverse pregnancy outcomes, including PTB [5-7].

Most studies assess GWG using IOM-based categories such as inadequate, adequate, and excessive, which provide clinically meaningful interpretation and are widely applied in obstetric practice. Previous research indicates that both insufficient and excessive GWG may be associated with an increased risk of PTB [8]. Evaluating GWG using these established categories, therefore, remains relevant for understanding its relationship with pregnancy outcomes and for guiding clinical decision-making.

Because weight gain patterns and their biological effects vary across BMI groups, restricting the analysis to women with normal pre-pregnancy BMI provides a more homogeneous population for evaluating this association [9]. Accordingly, this matched case-control study aimed to assess the association between GWG and PTB among women with normal pre-pregnancy BMI using IOM-based categorical classification of GWG.

## Materials and methods

Study design and setting

The matched case-control study was conducted between February 2022 and June 2024 at the Women and Children’s Hospital, a tertiary care centre in southeastern India. The institution predominantly serves a rural population and records 9,000-11,000 deliveries annually, with preterm births accounting for 8-14% and neonatal intensive care unit (NICU) admissions accounting for around 22% (unpublished data). As a regional referral centre, it manages a substantial proportion of high-risk obstetric cases from the adjoining districts. Women meeting the inclusion criteria were approached in the postnatal ward before discharge, and written informed consent was obtained. Clinical and obstetric data were obtained through medical records review and patient interviews.

Study procedure

Selection of Cases and Controls

**Figure 1:**
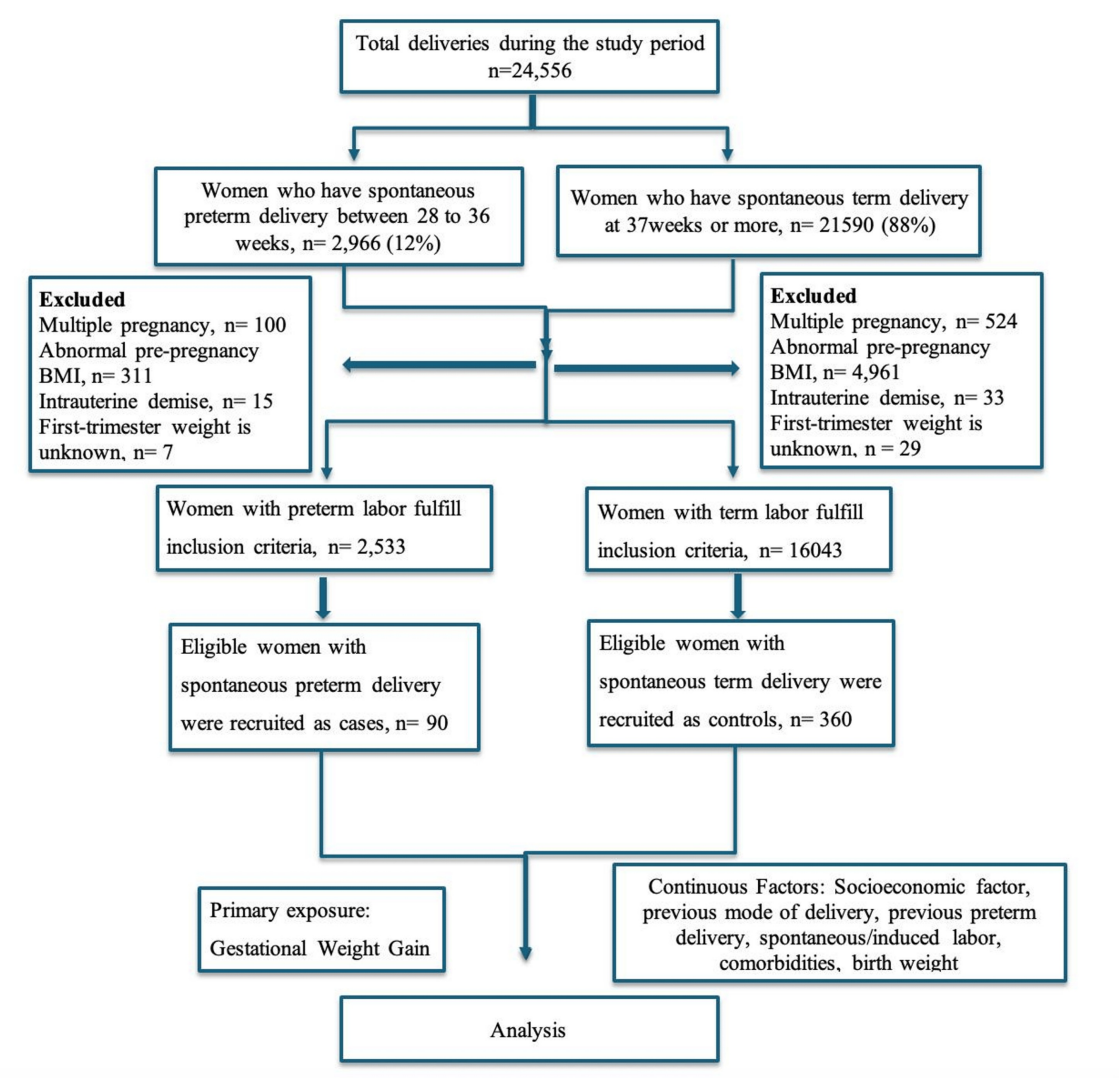
Flow diagram of study cohort recruitment.

The study population comprised women with a normal pre-pregnancy BMI ranging from 18.5 to 24.9 kg/m². Women who had spontaneous preterm delivery between 28 and 36 weeks of gestation were considered cases. Controls were women with spontaneous delivery at or beyond 37 completed weeks, matched for age (±2 years) and parity (primiparous or multiparous). Four controls were selected for each case, two recruited consecutively before and two after each case, yielding a 1:4 case-to-control ratio. Women were excluded if they had a history of antenatal intrauterine fetal death or if first-trimester weight records, essential for accurate GWG assessment, were unavailable. Figure 1 illustrates the details of the study cohort recruitment.

Exposure

The primary exposure variable in this study was GWG, defined as the difference between maternal weight at the time of delivery and the pre-pregnancy weight. In cases where pre-pregnancy weight was not documented, the earliest recorded weight before 14 weeks of gestation was used as a proxy, given that weight gain during the first trimester is typically minimal (approximately 100-200 grams) and considered negligible. GWG was categorised according to the IOM guidelines for women with a normal pre-pregnancy BMI: inadequate (<11.3 kg), adequate (11.3-15.8 kg), and excessive (>15.8 kg) [4]. These categories were used to examine the relationship between weight gain during pregnancy and the risk of preterm birth among women with a normal BMI.

Data Collection

Data were systematically extracted from patient medical records, and included maternal age, educational attainment, occupation, parity, gestational age at delivery, weight measurements taken pre-conception or at the first antenatal visit, and weight before delivery. Additionally, the presence of comorbid conditions such as anaemia, gestational diabetes mellitus, and hypertensive disorders of pregnancy was recorded. Additional variables included labour characteristics, mode of delivery, maternal and neonatal outcomes, and detailed obstetric history (prior term or preterm deliveries and associated complications). The dietary history variable was collected via questionnaires and coded as a binary covariate. Participants reporting exclusively vegetarian diets were coded as 'vegetarian', while those consuming both plant- and animal-based foods were coded as 'mixed diet'.

Sample Size Calculation

Based on previously published data, the prevalence of preterm birth among women with adequate GWG and a normal pre-pregnancy BMI is estimated to be 10.3% [10]. Assuming an increased risk of preterm birth associated with excessive GWG, reflected by an odds ratio of 2.5, and aiming for a statistical power of 80% at a 5% significance level, the required sample size was calculated to be 84 cases and 336 controls, adhering to a case-to-control ratio of 1:4.

Statistical analysis

Continuous variables, such as age and inter-delivery interval, were summarised as mean ± standard deviation (SD) or median with interquartile range (IQR), contingent upon the distribution of the data. These variables were compared between groups using either the Student’s t-test for normally distributed data or the Mann-Whitney U test for non-parametric data. Categorical variables, including socioeconomic status, education level, comorbidities, parity, and mode of delivery, were presented as frequencies and percentages and analysed using the chi-square test or Fisher’s exact test, as appropriate.

Covariates based on clinical relevance and existing evidence on determinants of preterm birth, consistent with a conceptual framework, were included in the multivariable model. We also included variables with a p-value < 0.10 on the univariate analysis. Conditional logistic regression was used to evaluate the association between GWG categories and the risk of preterm delivery, adjusting for matched variables (age and parity) and potential confounders such as socioeconomic status, comorbidities, history of previous preterm birth, and dietary history. Unadjusted odds ratios (ORs) were initially calculated in univariate analyses, followed by adjusted ORs, with 95% confidence intervals, from the multivariable regression.

Ethical approval

This study was conducted as per the ethical standards of the Institute's Scientific Advisory and Ethical Committee for human studies, following the 1964 Declaration of Helsinki and its later versions. The study was approved by the Institutional Ethics Committee for Observational Studies, Jawaharlal Institute of Postgraduate Medical Education and Research (Approval number: JIP/IEC/-OS/2022/196; dated: 18 August 2022).

## Results

Among 450 participants (90 (20%) cases and 360 (80%) controls), baseline characteristics such as maternal age, gravidity, height, pre-pregnancy weight, and inter-pregnancy interval were comparable. However, women with preterm cases had significantly lower gestational age at delivery (32.85 vs. 38.72 weeks, p < 0.001), higher pre-pregnancy BMI (22.13 vs. 21.71 kg/m², p = 0.042), and lower maternal weight before delivery (59.44 vs. 60.85 kg, p = 0.044). Birth weight was markedly reduced in cases (1.76 vs. 2.88 kg, p < 0.001). Overall, inadequate GWG and reduced gestational age were strongly associated with preterm birth, despite comparable baseline demographics (Tables 1, 2). Previous preterm delivery (5.6% vs. 1.4%, p = 0.016) and prior lower segment caesarean section (LSCS) (16.7% vs. 4.4%, p < 0.001) were more frequent among cases.

**Table 1:** Comparison of maternal characteristics by study groups (N = 450).

Variable	Control (n = 360), Mean ± SD	Case (n = 90), Mean ± SD	Mean difference (95% CI)	p-value
Gestational age at delivery (weeks)	38.72 ± 0.98	32.85 ± 2.31	5.88 (5.56 to 6.19)	<0.001
Maternal age (years)	26.33 ± 3.68	26.40 ± 3.72	-0.07 (-0.92 to 0.79)	0.873
Gravida	1.48 ± 0.50	1.48 ± 0.50	0.00 (-0.12 to 0.12)	1.000
Pre-pregnancy BMI (kg/m²)	21.71 ± 1.70	22.13 ± 1.89	-0.42 (-0.82 to -0.01)	0.042
Pre-pregnancy weight (kg)	52.03 ± 5.46	52.68 ± 6.03	-0.65 (-1.94 to 0.64)	0.323
Height (cm)	154.72 ± 5.96	154.11 ± 4.47	0.61 (-0.71 to 1.93)	0.365
Weight before delivery (kg)	60.85 ± 5.72	59.44 ± 6.56	1.41 (0.04 to 2.77)	0.044
Gestational weight gain (kg)	8.91 ± 1.86	6.77 ± 2.17	2.15 (1.70 to 2.59)	<0.001
Inter-pregnancy interval (years)	1.27 ± 1.45	1.38 ± 1.66	-0.10 (-0.45 to 0.24)	0.555
Birth weight (kg)	2.88 ± 0.94	1.76 ± 0.74	1.11 (1.03 to 1.19)	<0.001

**Table 2:** Distribution of categorical variables by group (N = 450). LSCS: lower segment caesarean section; IOM: Institute of Medicine.

Variable	Category	Control (n = 360) (80%)	Case (n = 90) (20%)	p-value
Socio-economic status	Low	50 (13.9%)	8 (8.9%)	0.059
	Middle	196 (54.4%)	42 (46.7%)	
	Upper	114 (31.7%)	40 (44.4%)	
Maternal education	Primary	61 (16.9%)	17 (18.9%)	0.018
	Secondary	56 (15.6%)	12 (13.3%)	
	Higher secondary	113 (31.4%)	15 (16.7%)	
	Graduate & above	130 (36.1%)	46 (51.1%)	
Previous preterm delivery	Yes	5 (1.4%)	5 (5.6%)	0.016
Previous vaginal delivery	Yes	172 (47.8%)	43 (47.8%)	1.000
Previous LSCS	Yes	16 (4.4%)	15 (16.7%)	<0.001
Parity	Primiparous	188 (52.3%)	47 (52.2%)	1.000
	Multiparous	172 (47.7%)	43 (47.8%)	
Hypertension in pregnancy	Yes	34 (9.4%)	11 (12.2%)	0.432
Gestational diabetes mellitus	Yes	32 (8.9%)	4 (4.4%)	0.164
Anaemia in pregnancy	Yes	103 (28.6%)	26 (28.9%)	0.958
Consanguinity	Yes	5 (1.4%)	1 (1.1%)	0.837
Gestational weight gain (IOM)	Adequate	225 (62.5%)	42 (46.7%)	0.008
	Less	111 (30.8%)	35 (38.9%)	
	Excessive	24 (6.7%)	13 (14.4%)	

The GWG (6.77 vs. 8.91 kg, p < 0.001) was lower among the cases. When classified according to the IOM criteria, it was significantly associated with preterm birth (p = 0.008), with inadequate and excessive gain being more frequent among cases. Table 3 presents the conditional logistic regression results. In bivariate analysis, both inadequate (OR: 1.70; 95% CI: 1.02-2.84; p = 0.041) and excessive GWG (OR: 2.90; 95% CI: 1.36-6.21; p = 0.006), and higher socioeconomic status (OR: 2.69; 95% CI: 1.08-6.71; p = 0.034), were significantly associated with the outcome. However, in multivariable analysis, only higher secondary maternal education (OR: 0.41; 95% CI: 0.18-0.98; p = 0.045) and previous LSCS (OR: 5.23; 95% CI: 1.87-14.67; p = 0.002) remained independently associated with preterm birth, after adjusting for other covariates.

**Table 3:** Bivariate and multivariable conditional logistic regression results (N = 450). LSCS: lower segment caesarean section; IOM: Institute of Medicine.

Variable	Category/scale	Bivariate OR (95% CI)	p-value	Multivariable OR (95% CI)	p-value
Gestational weight gain (IOM)	Less vs. adequate	1.70 (1.02-2.84)	0.041	1.51 (0.88-2.61)	0.138
	Excessive vs. adequate	2.90 (1.36-6.21)	0.006	1.98 (0.86-4.58)	0.110
Gestational weight gain (kg)	Per 1 kg	0.53 (0.45-0.63)	<0.001	-	-
Socioeconomic status	Middle vs. low	1.48 (0.62-3.51)	0.379	1.49 (0.56-3.98)	0.425
	Upper vs. low	2.69 (1.08-6.71)	0.034	2.00 (0.60-6.72)	0.260
Maternal education	Secondary vs. primary	0.76 (0.34-1.74)	0.521	0.92 (0.37-2.24)	0.849
	Higher secondary vs. primary	0.50 (0.23-1.08)	0.079	0.41 (0.18-0.98)	0.045
	Graduate+ vs. primary	1.35 (0.69-2.63)	0.386	1.00 (0.41-2.47)	0.998
Previous preterm delivery	Yes vs. no	4.53 (1.20-17.09)	0.026	3.86 (0.78-19.03)	0.097
Previous LSCS	Yes vs. no	5.53 (2.30-13.30)	<0.001	5.23 (1.87-14.67)	0.002
Dietary history	Yes vs. no	0.17 (0.03-1.00)	0.050	0.26 (0.04-1.89)	0.182
Parity	≥2 vs. 1	2.00 (0.18-22.06)	0.571	0.27 (0.02-3.89)	0.338
Pre-pregnancy BMI	Per 1 kg/m²	1.17 (1.01-1.34)	0.033	-	-
Inter-pregnancy interval	Per 1 year	1.28 (0.90-1.80)	0.167	-	-
Hypertension in pregnancy	Yes vs. no	1.35 (0.64-2.84)	0.425	-	-
Gestational diabetes mellitus	Yes vs. no	0.48 (0.17-1.40)	0.179	-	-
Anaemia in pregnancy	Yes vs. no	1.01 (0.60-1.71)	0.958	-	-
Consanguinity	Yes vs. no	0.80 (0.09-6.85)	0.839	-	-

## Discussion

In this case-control study of 450 participants, maternal age, gravidity, height, pre-pregnancy weight, and inter-pregnancy interval were comparable between cases and controls, indicating baseline similarity between groups. Women with PTB, however, had significantly lower gestational age at delivery and markedly reduced birth weight. Even as the GWG, both inadequate and excessive, was found to be associated with PTB on bivariate analysis, when adjusted for the potential confounders, it was not associated with PTB.

Women with preterm cases had lower GWG, and an observed U-shaped relationship with PTB, similar to the prior studies, reported an elevated risk of PTB with extremes of GWG [11,12]. Inadequate GWG might reflect the maternal undernutrition or impaired placentation, leading to low birth weight and fetal growth restriction, thereby increasing the risk of PTB. Excessive GWG may predispose to the metabolic and inflammatory changes that could trigger pathways resulting in PTB. These also suggest the need to align and adhere to the IOM-recommended GWG, as the deviation from them in either direction increases the risk of adverse outcomes, especially in women with normal pre-pregnancy BMI [3,4].

In the present study, inadequate GWG and lower gestational age were strongly associated with an increased risk of PTB. This finding is in line with previous reports demonstrating that women with insufficient GWG have a significantly higher likelihood of preterm delivery compared with those who achieve adequate weight gain during pregnancy [3]. Furthermore, subgroup analyses from their studies have shown that women aged 30-34 years and those who were underweight before pregnancy are at particularly high risk of PTB when GWG is inadequate.

However, these observations are partially inconsistent with some previous studies [13-15]. Notably, a large population-based study from South China provided a comprehensive evaluation of the association between GWG and PTB and reported variations in risk patterns. Such discrepancies may be attributable to differences in maternal age distribution and pre-pregnancy BMI across study populations, which could have influenced the observed associations.

Maternal education and prior obstetric events were demonstrated to be independently associated with PTB. Those with higher education were protective, suggesting improved health literacy and nutritional awareness influence maternal care-seeking behaviours, reducing the risk of PTB [16,17]. A history of LSCS significantly increased PTB risk, consistent with evidence linking uterine scarring and impaired cervical competence to prematurity [18,19]. The number of patients with prior PTB was small; this finding should therefore be interpreted cautiously in the context of well-established evidence from larger cohorts [20].

Strengths and limitations

This study’s strengths include its matched case-control design, which minimised confounding by age and parity, and restriction to women with normal BMI to reduce heterogeneity, allowing assessment of GWG as a risk factor for PTB. Comprehensive data collection from medical records and structured interviews, as well as conditional logistic regression to perform the matched analysis, could also be considered a strength. Certain limitations warrant consideration in the study. A key limitation is the use of IOM-based total GWG categories, which assume term delivery, potentially leading to misclassification in preterm cases. The alternative of calculating the rate per week also introduces difficulty in interpretation, as it is well known that weight gain is not linear and differs at each week of gestation, suggesting the need for population-based Z scores for gestational age to assess their role, especially in PTB. Another limitation is that the higher assumed effect size used in the sample size calculation, compared with the observed adjusted OR, may have limited power to detect smaller associations, which may have contributed to the null multivariable findings.

A potential recall bias for pre-pregnancy weight and the reliance on early pregnancy weight as a proxy for pre‑pregnancy weight may have introduced minor misclassification, although first‑trimester gain is typically negligible. Residual confounding from unmeasured factors such as physical activity, psychosocial stress, micronutrient intake, or environmental exposures cannot be excluded. Finally, conducting the single-centre study in a high-volume tertiary referral centre may limit the generalizability of the results.

Clinical implications

Routine antenatal care should document maternal weight and contextualise it against recommended ranges, enabling early identification of women at risk from inadequate and excessive gain. Integrating GWG surveillance into routine clinical practice could facilitate timely nutritional counselling, lifestyle interventions, and closer surveillance of high‑risk pregnancies. The protective effect of maternal education highlights the value of strengthening health literacy initiatives. At the same time, the association with prior caesarean delivery suggests that women with such histories may benefit from targeted preconception and antenatal counselling.

## Conclusions

In women with normal pre-pregnancy BMI, a U-shaped association between GWG and PTB was seen in unadjusted analyses but not after adjustment, highlighting the importance of adhering to recommended weight gain guidelines. Maternal education confers a protective effect, while a history of caesarean delivery significantly elevates the risk, reflecting an interplay of behavioural, biological, and obstetric factors on pregnancy outcomes. Focused interventions, such as nutrition and weight monitoring, increasing maternal health literacy, and specialised care for high-risk women, could prevent preterm deliveries and improve perinatal outcomes.
